# Gender-based equality in sport and women's presence in sport and sports leadership structures matrix 1.0 nation reports: a protocol for national and global annual report

**DOI:** 10.3389/fspor.2025.1598593

**Published:** 2025-08-26

**Authors:** Balsa Kascelan, Borko Katanic, Karuppasamy Govindasamy, Vlad Adrian Geantă, Alexandru Ioan Băltean, Milena Mitrovic, Bojan Masanovic

**Affiliations:** ^1^ Faculty of Law, University of Montenegro, Podgorica, Montenegro; ^2^ Montenegrin Sports Academy, Podgorica, Montenegro; ^3^ Department of Sports Recreation and Wellness, Symbiosis International (Deemed University), Hyderabad Campus, Modallaguda, Nandigama, India; ^4^ Faculty of Physical Education and Sport, Aurel Vlaicu University of Arad, Arad, Romania; ^5^ Doctoral School of Sport Science and Physical Education, Pitești University Center, National University of Science and Technology Politehnica Bucharest, Pitești, Romania; ^6^ Faculty for Sport and Physical Education, University of Montenegro, Niksic, Montenegro; ^7^ Western Balkan Sport Innovation Lab, Podgorica, Montenegro

**Keywords:** gender equality, women's leadership, country factsheets, monitoring, female, surveillance

## Abstract

This protocol was developed to facilitate the collection of up-to-date data and produce a comprehensive annual assessment of gender-based equality in sport and the representation of women in sports and leadership roles. Its goal is to provide evaluators with a standardized methodology for identifying and assessing key indicators related to gender equality and women's participation in sport at national, regional, and global levels. By focusing on a range of indicators, this protocol aims to highlight the current state of gender representation in sport and identify specific barriers to achieving equality. The protocol includes 19 sociodemographic indicators, accessible online, to characterize the demographic profile of participating countries. Additionally, it proposes 20 content indicators specifically focused on assessing women's presence in sport and leadership roles, as well as policies promoting gender equality. Each content indicator will be evaluated through separate analyses using a ten-point grading scale, allowing for a nuanced understanding of challenges and progress in this area. To ensure a dynamic connection between research and practice, the protocol encourages national evaluators to meet annually, producing national reports known as National Reports. These reports will offer detailed and ongoing assessments of gender equality in sport, with outcomes designed to inform policy development and advance gender equity in sports and leadership structures.

## 1 Introduction

Gender refers to the roles, behaviors, activities, and attributes that a society associates with masculinity and femininity (1). Gender equality, as defined by the United Nations, is the state in which women and men have equal rights and opportunities across all sectors of society. It is a condition where the diverse behaviors, aspirations, and needs of both women and men are equally valued (2).

Achieving gender equality involves attaining equal representation across various measurable outcomes, such as equal access and participation, equal representation in leadership, equal pay and rewards, and equal media coverage and visibility (3). Significant progress has been made in gender equality in sports globally. For example, at the Tokyo 2020 Olympics, women made up 48.8% of participants (4), bringing the event close to achieving gender parity. Additionally, the presence of women in leadership roles within international sports federations has increased, with women now holding about 21% of leadership positions (5). Despite this progress, challenges remain. Achieving full gender equality is an ongoing struggle, and maintaining these gains requires continuous effort and commitment.

Gender inequality brings a number of interconnected disadvantages. Women and girls often have limited access to education, which affects their opportunities in later life. This limits their potential, depriving companies and economies of valuable skills and talents. As a result, gender inequality contributes to economic inefficiency. Moreover, male-dominated power structures lead to social injustice, which can result in health consequences, violence, political exclusion, and social instability. Given these challenges, gender equality is a core value of the European Union (EU), embedded in its legal frameworks, policies, and strategies (6). The EU has long been committed to promoting gender equality in sports and closing gender gaps. Through programs such as Erasmus + Sport, the EU supports projects and partnerships that aim to reduce gender inequality in sports by fostering women's leadership, participation, and inclusion in various sectors. One such initiative, the SWOST project, inspired the authors of this study to develop a gender equality monitoring system. This system aims to provide updated data on gender equality in sports, motivating ongoing progress and keeping the goal of gender equality at the forefront.

While literature emphasizes the need for an organized monitoring system, no universally accepted framework currently exists, and many countries do not implement such systems. Several institutions monitor gender equality to identify gaps and inequalities, providing a foundation for shaping policies that promote gender equality. However, monitoring efforts are often underdeveloped or limited, and their effectiveness varies depending on political will, available resources, and the quality of data collected. The European Union's Gender Equality Index (7) provides valuable data, but it is not sufficiently detailed for local-level application. A comprehensive and consistent monitoring system is essential, followed by concrete actions aimed at reducing gender inequality.

The goal of this study is to develop a methodology that will provide a reliable foundation for tracking specific indicators over time, allowing for the assessment of changes in gender equality at national, regional, and global levels. This will involve collecting relevant data on gender-based equality in sports, women's participation in sport, and women's presence in sports leadership roles, along with the barriers to achieving these outcomes in each country. Periodic evaluations will inform the creation of National Reports, which will then be consolidated into a global report. National Reports will provide actionable insights to inform sports policies and promote structural gender equity in leadership and participation.

## 2 Materials and methods

A team of content experts has been assembled to develop a new study protocol designed to serve as a reliable tool for collecting up-to-date data on gender-based equality in sport and the presence of women in sports and leadership structures. This protocol, built around a set of specific indicators to which grades will be assigned, is supported by the SWOST (Sport Without Stereotypes) project team under the Erasmus + Sport program (Project No: 622774-EPP-1-2020-1-IT-SPO-SCP).

The study protocol will encourage national evaluators (researchers) to meet annually, review the most relevant evidence related to gender equality in sport and women's representation in sports leadership, and produce national reports (National Reports). These reports will ensure dynamic and effective connections between research and practice. The National Reports will be crucial in guiding discussions that promote actions aimed at enhancing gender equality and increasing women's representation in both sport and leadership positions, contributing to more inclusive and equitable environments within the sports industry.

The protocol draws on the authors’ extensive experience with leading methodologies for assessing gender equality globally (8–10). Its purpose is to support individuals and organizations dedicated to promoting gender equality in sport and women's leadership in sport. The primary outcome of this study protocol will be the National Reports, created through a consistent, transparent process of gathering and compiling the best available evidence. These factsheets, available in both concise and detailed formats in English, will be shared through media and public awareness campaigns, knowledge exchanges at scientific conferences and workshops, and collaborations with relevant stakeholders.

This protocol includes 19 sociodemographic indicators that are publicly available online and will be used to assess the characteristics and demographic profiles of the participating countries. These indicators are: country; total population; urban population; female population percentage; human capital index; GDP per capita; GDP growth; unemployment rate; literacy rate (overall and by gender); educational attainment (percentage with tertiary education overall and by gender); labor force participation (overall and by gender); government spending on education; government spending on sports and recreation; internet usage; life expectancy; physical activity prevalence; workforce gender distribution; age distribution (youth and adult populations); and socioeconomic status (poverty rate or income inequality index) (Table 1).

**Table 1:** Country's demographic profile.

Questions (unit)	Definition	Reason for inclusion	Application in protocol
Country (–)	Name of the country for which data are provided	Identifies the research entity	Specifies the national context of evaluation
Total population (number of people)	Total number of inhabitants	Basis for ratios and weightings	Used to normalize indicators
Urban population (%)	% of population living in urban areas	Reflects differences in infrastructure availability	Adjusts protocol for urban vs. rural settings
Female population percentage (%)	% of population that are women	Key for assessing gender dynamics	Ensures gender-specific analysis
Human capital index (number between 0 and 1)	Value between 0 and 1 reflecting education, health, and skills	Measures development level and human resource potential	Provides broader socio-economic context
GDP per capita (current US$)	GDP in current USD per person	Indicator of economic development	Informs financial capacity for protocol implementation
GDP growth (annual %)	Annual GDP growth rate	Measures economic trend	Provides temporal context for evaluation
Unemployment rate, total (% of total labor force)	% of working-age population without employment	Affects socio-economic status and time for activities	Weights access to sports facilities
Literacy rate, adult total and by gender (% of people ages 15 and above)	% of adults aged 15+ who can read and write (overall and by gender)	Crucial for understanding materials and communication	Tailors complexity of information
Educational attainment, percentage with tertiary education overall and by gender (%)	% of population with tertiary education (overall and by gender)	Indicator of educational structure and gender gaps	Used to plan educational and promotional measures
Labor force participation, overall and by gender (% of people ages 18 and above)	% of population aged 18+ in the workforce (overall and by gender)	Shows economic engagement and gender disparities	Informs resource availability for evaluators
Government expenditure on education, total (% of government expenditure)	% of total government expenditure on education	Indicator of educational policy priority	Contextualizes investment in human capital
Government expenditure on sporting and recreational services, total (% of government expenditure)	% of total government expenditure on sports and recreation	Reflects support for sports programs	Assesses resources available for sports infrastructure
Individuals using the Internet (% of population)	% of population using the internet	Determines feasibility of digital engagement and data collection	Guides online components of the protocol
Life expectancy at birth, total (years)	Average number of years a newborn is expected to live	Proxy for health and living standards	Benchmark for health-related protocol targets
Physical activity prevalence (%)	% of adults not meeting physical activity guidelines	Shows baseline activity level	Helps set targets for increasing activity
Workforce gender distribution (%)	% of males and females in the workforce	Reflects gender structure of the labor market	Ensures gender-balanced analyses
Age distribution, youth and adult populations (%)	% of population by age groups (youth and adults)	Measures demographic structure	Adjusts protocol for different age groups
Socioeconomic status, poverty rate or income inequality index (% or number between 0 and 1)	Poverty rate or Gini index of income inequality (as % or 0–1 index)	Reflects economic inequalities	Targets interventions in socially excluded groups

The protocol also identifies 20 content indicators focused specifically on women's participation in sport and sports leadership, as well as policies for advancing gender equality. These include: Women's participation in competitions; Women's presence in the Olympic team; Women's presence in Ministry responsible for sport staff; Women's presence in National Olympic Committee leadership; Women's presence in National Paralympic Committee leadership; Women's presence in national sport federations management structures; Woman as a president of a national sport federation; Women as board members in a national sport federation; Women as assembly members in national sport federations; Women in coaching roles; Women in elite coaching roles; Women in sports referee roles; Women as delegates; Women's presence in medical staff personnel; Women's presence in sports media staff; Written policy or legislation on gender equality; Statutes revised and adapted from a gender perspective; Availability of leadership training for women; Clear gender-friendly recruitment procedures; Policy for combating gender-based violence (Table 2).

**Table 2:** GBES-WPSLS Matrix 1.0 Indicators and Benchmarks Used to Guide the Grade Assignment Process.

Indicator	Benchmark
Women's Participation in Competitions	% of women participating in national and international sports competitions, including elite, amateur, and youth levels.
Women's Presence in the Olympic Team	% of women represented in national Olympic teams across various sports.
Women's Presence in Ministry Responsible for Sport Staff	% of women in staff roles within government ministries or departments responsible for sports and recreation.
Women's Presence in National Olympic Committee Leadership	% of women in leadership roles (e.g., president, vice-president, executive director) within National Olympic Committees (NOCs).
Women's Presence in National Paralympic Committee Leadership	% of women in leadership roles within National Paralympic Committees (NPCs).
Women's Presence in National Sport Federations Management Structures	% of women in senior management or executive roles within national sport federations (e.g., directors, managers).
Women as President of a National Sport Federation	% of women serving as presidents of national sport federations.
Women as Board Members in a National Sport Federation	% of women serving as board members within national sport federations.
Women as Assembly Members in National Sport Federations	% of women serving as assembly or governing body members in national sport federations.
Women in Coaching Roles	% of women in coaching roles at various levels of sport, from grassroots to elite.
Women in Elite Coaching Roles	% of women in coaching roles at the elite or professional level, including national and international team coaches.
Women in Sports Referee Roles	% of women in officiating roles in sports, including referees, umpires, and match officials at all levels of competition.
Women as Delegates	% of women serving as official delegates or representatives at national or international sports events, conferences, or governing bodies.
Women's Presence in Medical Staff Personnel	% of women in medical roles (e.g., physicians, physiotherapists, nutritionists) within sports teams or federations.
Women's Presence in Sports Media Staff	% of women in sports media roles, including journalists, reporters, analysts, and broadcasters.
Written Policy or Legislation on Gender Equality	% of national sports organizations that have formal written policies or legislation addressing gender equality in sport, including participation and leadership.
Statutes Revised and Adapted from a Gender Perspective	% of national sports federations or committees that have revised their statutes or regulations with a gender perspective in mind, ensuring equal opportunity for women.
Availability of Leadership Training for Women	% of national or international sport organizations offering specific leadership training or development programs for women in sport.
Clear Gender-Friendly Recruitment Procedures	% of sport organizations with formal, clear, and gender-friendly recruitment procedures to ensure equal opportunity for women in sport roles.
Policy for Combating Gender-Based Violence	% of national sport federations or organizations with active, clear policies or programs aimed at combating gender-based violence in sports environments.

The authors of the protocol plan to regularly gather input from stakeholders to enhance and update the list of indicators annually.

The 20 content indicators were selected based on: (1) relevance to gender equality monitoring in sport, (2) precedence in international frameworks (e.g., EU Gender Equality Index) (7), (3) findings from the State of PLAY National Framework Analysis—one of the key outputs of the SWOST project (11) and (4) feasibility of data collection across countries. The process was further informed by the authors’ own methodological expertise—acquired through previous development of gender-equality assessment tools and the other experience gained on the SWOST project—and by consultations with academic experts in sport science, gender studies, and policy analysis, thereby ensuring the indicators’ applicability, validity, and operational feasibility.

Additionally, they will conduct external evaluations to assess the protocol's inputs, outputs, and both immediate and long-term outcomes.

To assess each of the content indicators, a separate analysis will be conducted using a six-point grading scale (5 = excellent; 4 = very good; 3 = good; 2 = fair; 1 = poor; 0 = lacking reliable information). The grades will be determined based on data from scientific articles published within the last 10 years, as well as secondary sources such as government and nongovernment reports and relevant online content from the specified period. To reduce bias and ensure consistency, sources will be assessed according to a reliability hierarchy: (1) peer-reviewed scientific publications, (2) official government and intergovernmental documents, (3) reports by recognized non-governmental organizations, and (4) online media or web content, which will be cross-verified where possible. Evaluators are instructed to prioritize higher-tier sources and to document the rationale for using lower-tier materials when necessary. The findings will then be synthesized, and the grading process will be finalized using the framework outlined in Table 3. Each indicator will be thoroughly discussed until a consensus on the grade is reached among the evaluators. While evaluators are expected to reach consensus on the grade for each indicator, a structured conflict resolution procedure is in place. In cases of disagreement, a two-step resolution process will be used: (1) evaluators will revisit the evidence and justify their grading using a standardized justification form; (2) if disagreement persists, a third independent evaluator will be invited to provide an assessment, and the final grade will be determined by majority decision.

**Table 3:** GBES-WPSLS Matrix 1.0 Grading System.

Grade	CI	Description
5	40.1%–50%	Excellent
4	30.1%–40%	Very good
3	20.1%–30%	Good
2	10.1%–20%	Fairly good
1	0.1%–10%	Poor
0	0	Without reliable information

CI, class intervals represent the range or difference between the upper and lower class limits of a given data set.

The present protocol adopts a binary operationalization of gender (women and men), which aligns with the structure of most current sports governance systems and available data sources. While the authors acknowledge the importance and existence of transgender and gender non-conforming individuals in sport, the scope of this study is limited to cisgender categories due to the availability, comparability, and consistency of existing datasets. This does not imply exclusion, but rather reflects a pragmatic decision for standardization in cross-country comparisons. Future iterations of the protocol may expand to incorporate more inclusive gender categories as data collection practices evolve.

The grading thresholds applied in this protocol—such as the definition of “excellent” performance corresponding to at least 40.1% representation—are grounded in the theoretical framework proposed by Kanter (12), who emphasized that true equality between groups is achieved when each gender attains a minimum threshold of 40% participation. This principle has also been adopted in institutional practice. For instance, the European Commission (13) set explicit targets requiring a minimum of 40% representation of both women and men on the boards of national sports organizations, expert working groups in sport, and among athletes and coaches. These thresholds provide a normative benchmark for evaluating structural gender balance and inform the cut-off points used in our grading scale.

The reliability of content analysis in this study on Gender-Based Equality in Sport and Women's Presence in Sport and Sports Leadership Structures will involve the operationalization of key concepts related to gender equality, women's participation, and leadership roles in sport. This includes training coders to accurately implement these concepts, as well as assessing coder reliability throughout the evaluation process (14). To address potential biases, the inter-rater reliability will be calculated using Cohen's kappa coefficient (κ), as recommended by McHugh (15). Research articles will be sourced from electronic databases such as SportDiscus, Scopus, PubMed/MEDLINE, and Web of Science. Additional resources like Open Access Theses and Dissertations (OATD), the Networked Digital Library of Theses and Dissertations (NDLTD), and Google will be used to find theses, dissertations, and other relevant documents or online content to assess the indicators. The full search syntax for each database will be detailed in Table 4.

**Table 4:** Full search syntax used for each database.

Database	Search syntax
Scopus	title-abs-key(“gender equality” OR “women in sport” OR “female in sport” OR “women leadership” OR “female leadership” OR “women athletes” OR “female athletes” OR “gender equity” OR “sports leadership”) AND title-abs-key([country])
Web of Science, SportDiscus (through EBSCOhost)	(“gender equality” OR “women in sport” OR “female in sport” OR “women leadership” OR “female leadership” OR “gender equity”) AND (“women athletes” OR “female athletes” OR “sports leadership”) AND [country]
Open Access Theses and Dissertations (OATD), Networked Digital Library of Theses and Dissertations (NDLTD)	(“gender equality” OR “women in sport” OR “female in sport” OR “women leadership” OR “female leadership” OR “gender equity”) AND (“women athletes” OR “female athletes” OR “sports leadership”) AND [country]
Google	"gender equality” OR “women in sport” OR “female in sport” OR “women leadership” OR “female leadership” OR “sports leadership” AND “women athletes” OR “female athletes” AND [country]

You can replace [country] with the name of a specific country or region depending on your research focus.

**Figure 1:**
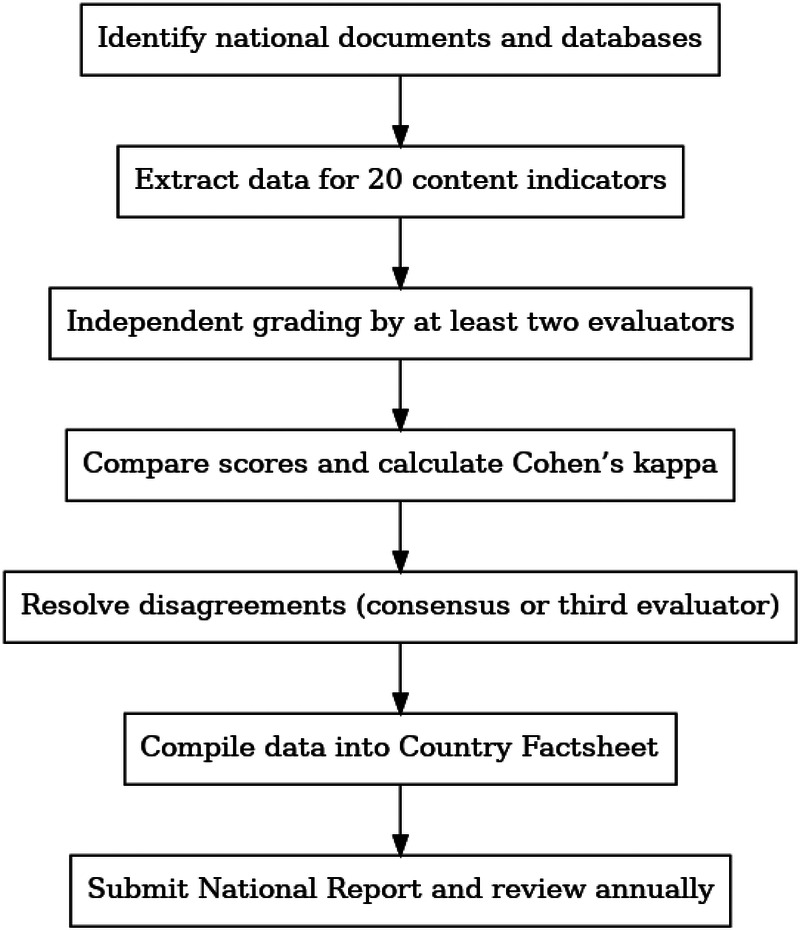
Step-by-step protocol for national-level data collection, indicator grading, consensus building, and reporting within the Gender-Based Equality in Sport Matrix 1.0 framework.

The overall evaluation process is summarized in the flowchart below (Figure 1), which outlines the main steps for national teams from data identification to final reporting.

## 3 Expected results

This protocol is designed to enable evaluators to gather current data and produce a comprehensive annual assessment through specific indicators, ensuring that the results reflect the most relevant evidence related to gender equality in sport and the presence of women in sports leadership roles across countries. It will also identify specific barriers women face in participating in sports or assuming leadership positions.

Evaluators will assess the results by comparing their grades, followed by calculating the initial inter-judge agreement using Cohen's kappa coefficient (κ). After reviewing all available data, evaluators will seek additional information to maximize inter-judge agreement, aiming for a kappa value of 1, indicating perfect agreement. A high level of intercoder reliability will confirm that all coders are thoroughly familiar with the coding protocol and codebook.

The expected results will be presented as scores (0–5) for each content indicator, awarded based on the quality and quantity of selected documents (scientific articles, governmental and nongovernmental reports, and online content). For each Country Factsheet, 20 content indicators will be reviewed to assess the status of gender equality in sport and women's presence in sports leadership. A total average score for these 20 indicators will be calculated, providing a snapshot of the status of gender equality and women's leadership in sports within each country.

Additionally, this section will outline the primary target audiences and the expected outcomes of the study protocol. The main audiences for the National Reports are sports organizations, policymakers, and educators whose work influences gender equality and the inclusion of women in sports and leadership roles. Media outlets are also considered key stakeholders, as their role in raising public awareness and promoting gender equality in sports is vital. While the general public may be indirectly impacted through media coverage, the protocol's core goal remains to inform practitioners, policymakers, and educators.

The dissemination of the National Reports will grow through social media campaigns and annual releases, focusing on recommendations to improve gender equality in sport and enhance the presence of women in leadership roles within sports organizations.

Immediate outcomes will include increased awareness among decision-makers about the importance of gender equality in sport, the contribution of the National Reports to raising awareness, and media engagement to highlight the importance of women's representation in sports. This will also involve the continuous engagement of relevant organizations advocating for policy and program changes to enhance opportunities for women in sports. Furthermore, the National Reports will be recognized as a reliable source of data regarding gender equality in sports and an advocate for women athletes and leaders.

Intermediate outcomes will focus on creating policies, programs, and campaigns by government and non-government stakeholders aimed at increasing opportunities for women in sports leadership and participation. Long-term outcomes will be measured by the percentage of women participating in competitive sports and holding leadership roles, reflecting the success of policies and programs promoting gender equality and women's representation in sports leadership.

## 4 Limitations

While this protocol offers a structured framework for detecting and quantifying equality across socio-demographic groups, it is not without limitations. First, the focus on equality—operationalized through proportional representation—does not guarantee equity, which requires tailoring resources and access to group-specific needs rather than distributing them identically. Consequently, participants who appear equally represented may still face unequal opportunities due to socio-economic status, educational background, or cultural barriers (16). Second, the current binary gender categorization (male–female) overlooks the complex, intersectional dimensions of identity described by Crenshaw (17), including race, class, sexual orientation, and other axes of marginalization. By not accounting for these overlapping inequalities, the protocol cannot fully capture or address compounded disadvantage. We regard these shortcomings as areas for future enhancement, envisioning subsequent iterations that introduce weighted adjustments for socio-economic factors and expand gender classification to an intersectional model in line with contemporary scholarship. In the next phase, we will pilot test socia-economic weighting adjustments to evaluate their feasibility before extending to a full intersectional framework.
